# Supplementary material for: Genome-scale reconstruction of Gcn4/ATF4 networks driving a growth program
Source: PLoS Genet. 2020 Dec 30;16(12):e1009252. doi: 10.1371/journal.pgen.1009252 (PMC7773203; doi:10.1371/journal.pgen.1009252)
Supplement: S1 Fig — Correlation plots between replicates of the RNA sequencing data, from both WT and Δgcn4 cells, for the indicated media conditions. Normalised read counts of the RNA sequencing data are plotted. Replicates shows good correlation with a Pearson correlation coefficient, R = 0.9. (PDF) [file pgen.1009252.s001.pdf]

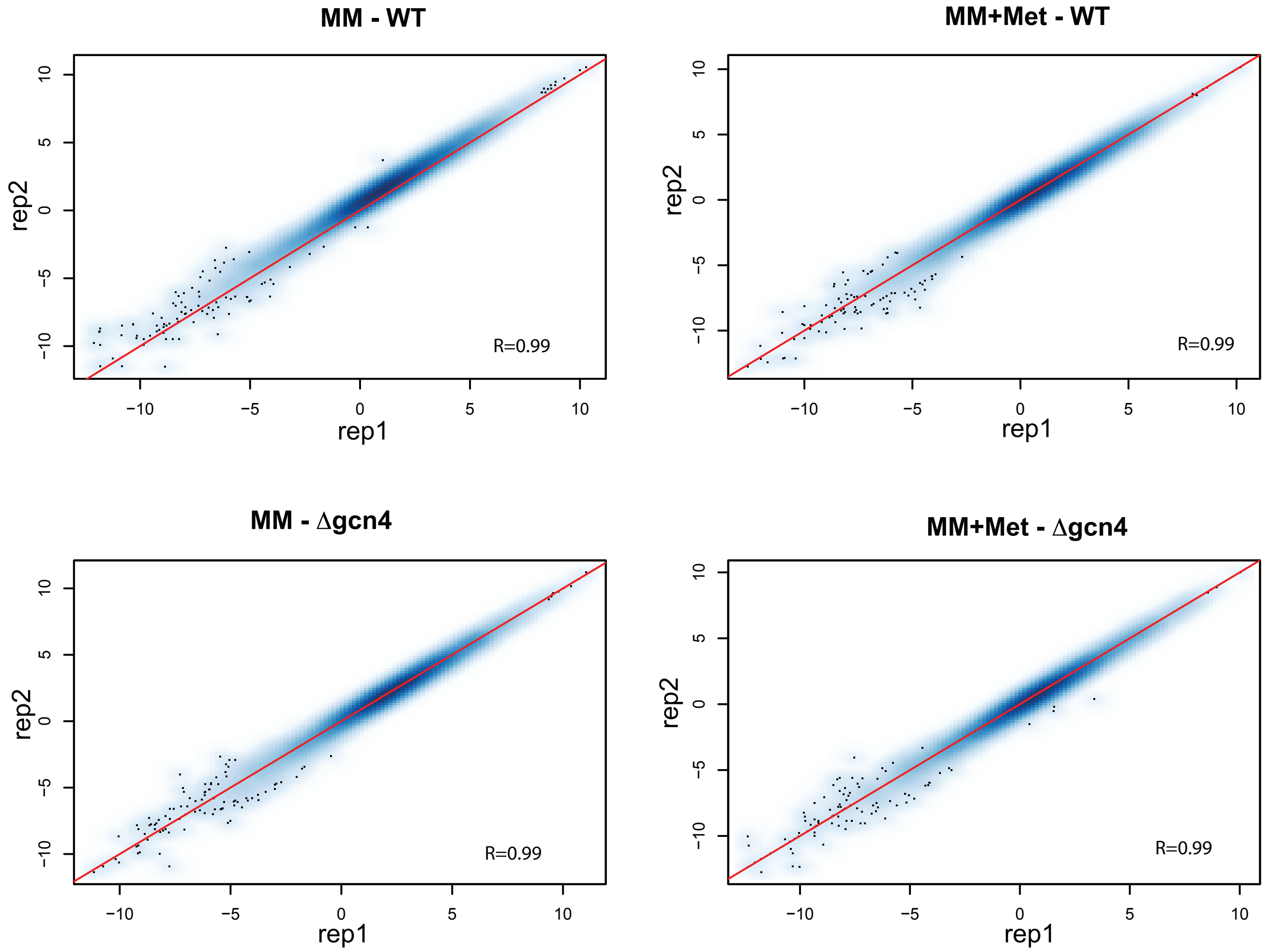

Supplementary Figure 1: RNA-seq correlation plots
Correlation plots between replicates of the RNA sequencing data, from both WT and Δgcn4 cells, for the indicated media conditions. Normalised read counts of the RNA sequencing data are plotted. Replicates shows good correlation with a Pearson correlation coefficient, R = 0.9.
