# Supplementary material for: Genome-scale reconstruction of Gcn4/ATF4 networks driving a growth program
Source: PLoS Genet. 2020 Dec 30;16(12):e1009252. doi: 10.1371/journal.pgen.1009252 (PMC7773203; doi:10.1371/journal.pgen.1009252)
Supplement: S2 Fig — A. Experimental design to study a growth program triggered by methionine. B. A volcano plot showing differentially expressed genes in MM+Met, relative to MM. Genes that are upregulated and downregulated in MM+Met (fold change of ≥ 1.5, p-value cut off of 10−4) are highlighted in red and blue respectively. C. A bar plot showing the most significantly enriched GO categories of the genes either induced or downregulated by methionine. The GO terms shown here are significantly enriched terms with the corrected p-value < 0.05 (hypergeometric test, Bonferroni Correction) (also see S3 Data for complete GO analysis results). For this, the numbers of genes induced (to the number of genes in that category) are also indicated within each bar. D. Heat maps showing the transcriptional induction of anabolic genes and translation related genes altered by methionine, i.e. in MM+Met relative to MM. Comparisons are made in two different carbon sources where methionine was added, glucose (this study) and lactate medium [5] (Also see S2 Data). (PDF) [file pgen.1009252.s002.pdf]

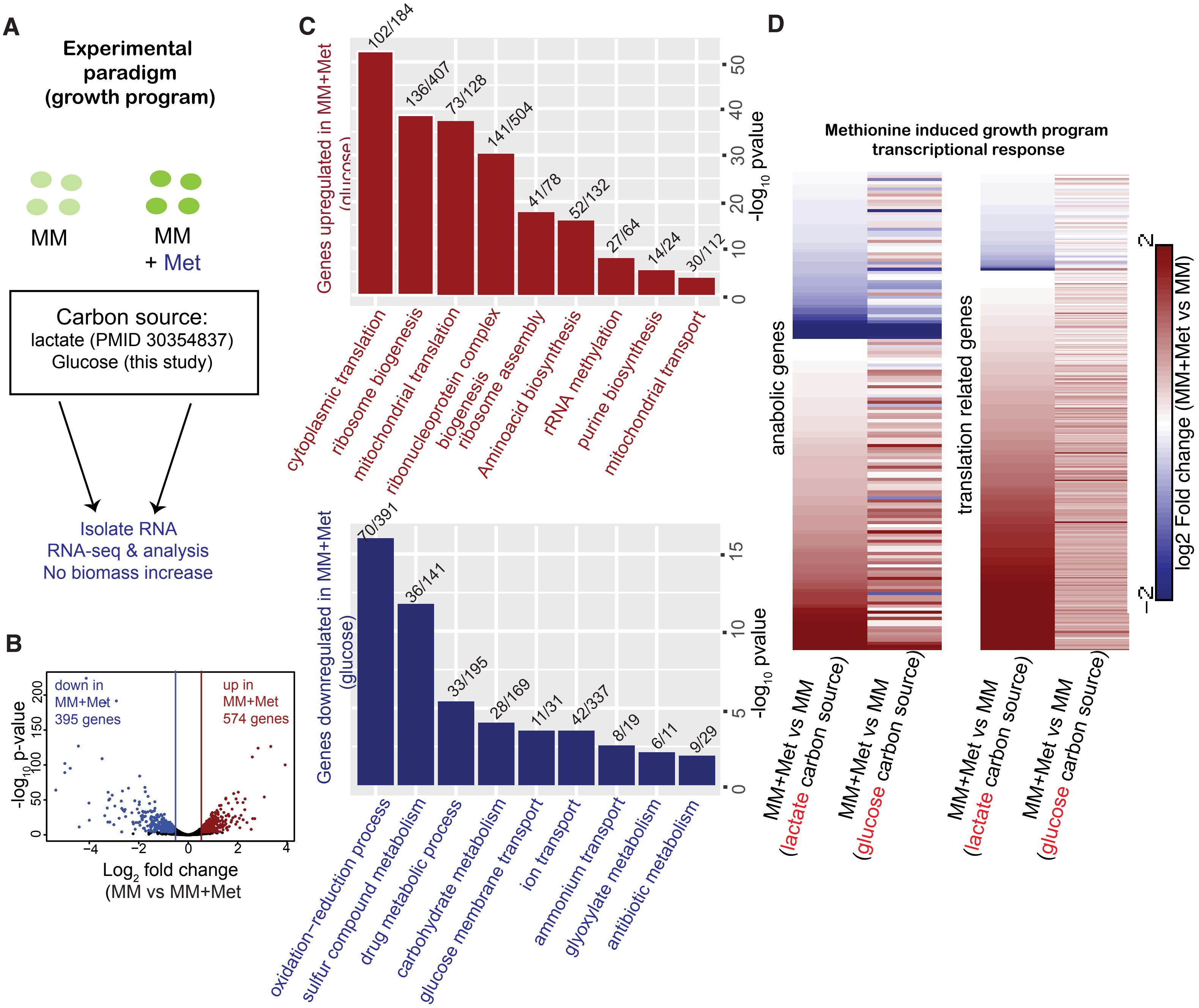

Supplementary Figure 2: Methionine induces a conserved transcriptional growth program irrespective of carbon sources

A. Experimental design to study a growth program triggered by methionine.

B. A volcano plot showing differentially expressed genes in MM+Met, relative to MM. Genes that are upregulated and downregulated in MM+Met (fold change of ≥ 1.5, p-value cut off of 10^{-4}) are highlighted in red and blue respectively.

C. A bar plot showing the most significantly enriched GO categories of the genes either induced or downregulated by methionine. The GO terms shown here are significantly enriched terms with the corrected p-value < 0.05 (hypergeometric test, Bonferroni Correction) (also see Supplementary WS3 for complete GO analysis results). For this, the numbers of genes induced (to the number of genes in that category) are also indicated within each bar.

D. Heat maps showing the transcriptional induction of anabolic genes and translation related genes altered by methionine, i.e. in MM+Met relative to MM. Comparisons are made in two different carbon sources where methionine were added, glucose (this study) and lactate medium [5] (Also see Supplementary WS2).
