# Supplementary material for: Genome-scale reconstruction of Gcn4/ATF4 networks driving a growth program
Source: PLoS Genet. 2020 Dec 30;16(12):e1009252. doi: 10.1371/journal.pgen.1009252 (PMC7773203; doi:10.1371/journal.pgen.1009252)
Supplement: S3 Fig — (Left panel) Quantitative LC/MS/MS based targeted metabolic flux measurements, using N15 ammonium sulfate labeling, to estimate nitrogen flux towards new amino acid synthesis in MM+Met compared to MM. The experiment was performed as illustrated in the schematic flow diagram. Data shown are the average of two biological replicates (with technical duplicates), with standard deviation. (Right panel) Quantitative LC/MS/MS based targeted metabolic flux measurements, using C13 glucose labeling, to estimate carbon flux towards new nucleotide synthesis in MM+Met compared to MM. Data shown are the average of two biological replicates (with technical duplicates), with standard deviation. * p-value < 0.05, ** p-value <0.01. (PDF) [file pgen.1009252.s003.pdf]

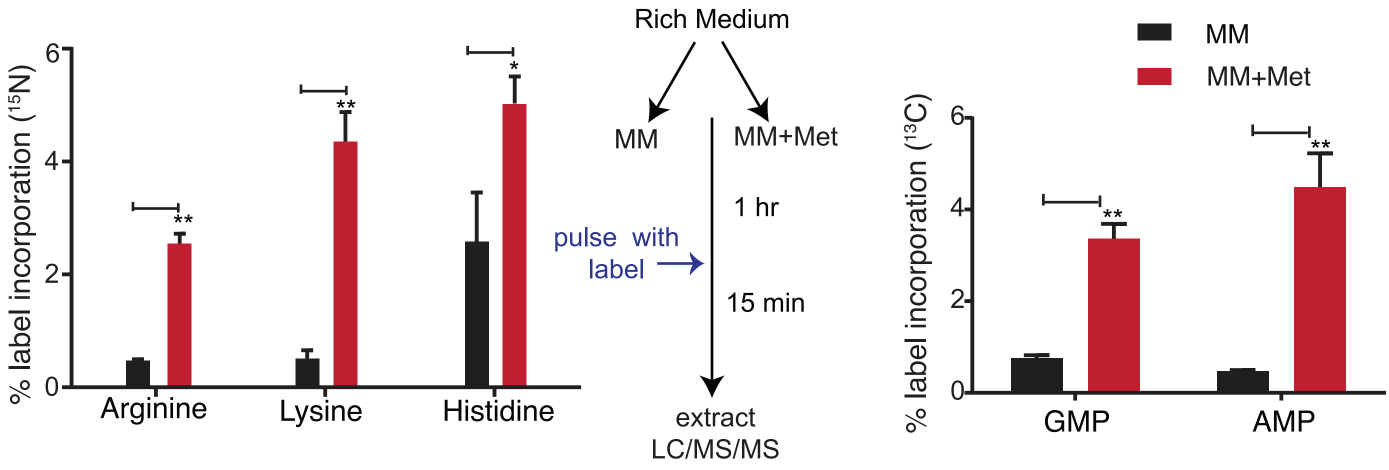

Supplementary Figure 3: Quantitative LC-MS/MS based metabolic flux measurements to establish the methionine induced anabolic program.

(Left panel) Quantitative LC/MS/MS based targeted metabolic flux measurements, using N^{15} ammonium sulfate labeling, to estimate nitrogen flux towards new amino acid synthesis in MM+Met compared to MM. The experiment was performed as illustrated in the schematic flow diagram. Data shown are the average of two biological replicates (with technical duplicates), with standard deviation.

(Right panel) Quantitative LC/MS/MS based targeted metabolic flux measurements, using C^{13} glucose labeling, to estimate carbon flux towards new nucleotide synthesis in MM+Met compared to MM. Data shown are the average of two biological replicates (with technical duplicates), with standard deviation. * p-value < 0.05, ** p-value <0.01.
