# Supplementary material for: Genome-scale reconstruction of Gcn4/ATF4 networks driving a growth program
Source: PLoS Genet. 2020 Dec 30;16(12):e1009252. doi: 10.1371/journal.pgen.1009252 (PMC7773203; doi:10.1371/journal.pgen.1009252)
Supplement: S4 Fig — WT cells show better growth when supplemented with methionine. Deletion of Gcn4 lead to severely reduced growth in the presence of methionine. Also see Fig 1A. (PDF) [file pgen.1009252.s004.pdf]

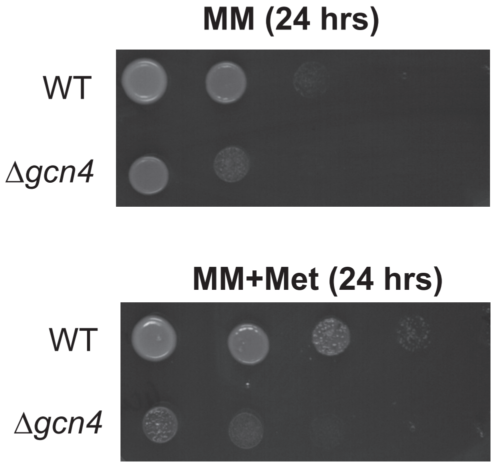


**Supplementary Figure 4: Serial dilution based growth assay, comparing WT and Δgcn4 cells growing in the presence or absence of Methionine.**
WT cells show better growth when supplemented with methionine. Deletion of Gcn4 lead to severely reduced growth in the presence of methionine. Also see Figure 1A.
