# Supplementary material for: Genome-scale reconstruction of Gcn4/ATF4 networks driving a growth program
Source: PLoS Genet. 2020 Dec 30;16(12):e1009252. doi: 10.1371/journal.pgen.1009252 (PMC7773203; doi:10.1371/journal.pgen.1009252)
Supplement: S5 Fig — A. A representative western blot for Gcn4-HA (Gcn4 tagged with the HA epitope at the endogenous locus) expression in the indicated medium. Gcn4 protein levels are high in MM+Met. Abbreviations: RM—rich medium, MM—minimal medium without amino acids and with glucose as a carbon source, and MM+Met—minimal medium without amino acids and with glucose as a carbon source, supplemented with 2mM methionine. B. (left panel) A volcano plot comparing transcript expression in WT with Δgcn4 cells (grown in MM+Met). Genes induced with a fold change cut-off of ≥ 2, and p-value ≤ 10−4 are shown in red, and the downregulated transcripts (similar cut-off parameters) are shown in blue. The numbers of differentially expressed transcripts are also indicated. (right panel) A volcano plot comparing transcript expression in WT with Δgcn4 cells (grown in MM. Genes induced with a fold change cut-off of ≥ 2, and p-value ≤ 10−4 are shown in red, and the downregulated transcripts (similar cut-off parameters) are shown in blue. The numbers of differentially expressed transcripts are also shown. C. Correlation plots between normalised read counts of the ChIP sequencing data, from cells growing in different medium. Normalised read counts of 100000 randomly selected positions from the genome are plotted. ChIP data show enrichment/DNA binding for Gcn4 only in methionine supplemented conditions. Replicates show good correlation with a Pearson correlation coefficient, R = 0.9. (PDF) [file pgen.1009252.s005.pdf]

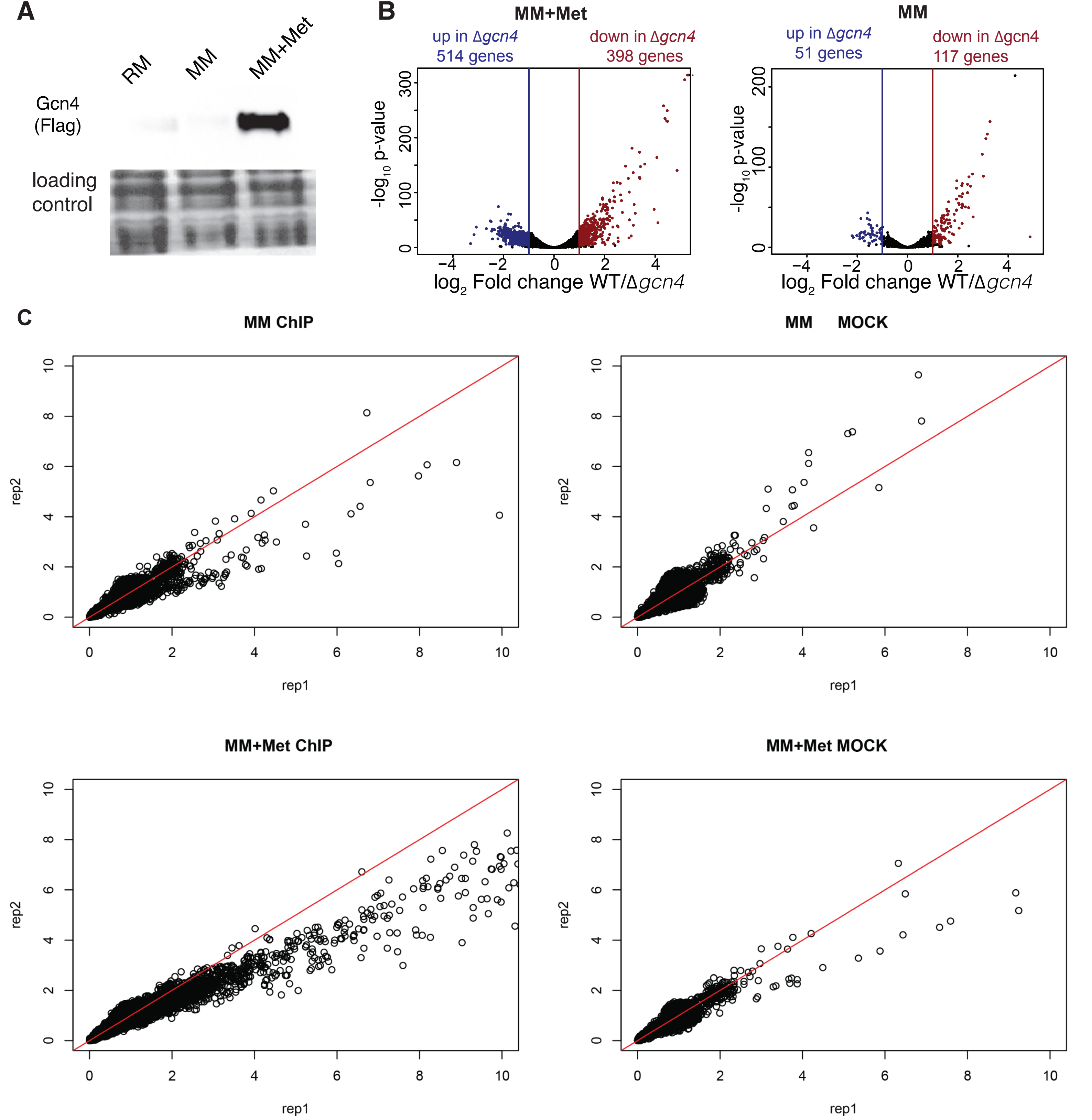

Supplementary Figure 5: Gcn4 protein expression, effect of loss of Gcn4 in MM and MM+Met, and Gcn4 ChIP-seq correlation plots

A. A representative western blot for Gcn4-HA (Gcn4 tagged with the HA epitope at the endogenous locus) expression in the indicated medium. Gcn4 protein levels are high in MM+Met. Abbreviations: RM - rich medium, MM - minimal medium without amino acids and with glucose as a carbon source, and MM+Met - minimal medium without amino acids and with glucose as a carbon source, supplemented with 2mM methionine.

B. (left panel) A volcano plot comparing transcript expression in WT with Δgcn4 cells (grown in MM+Met). Genes induced with a fold change cut-off of ≥ 2, and p-value ≤ 10^{-4} are shown in red, and the downregulated transcripts (similar cut-off parameters) are shown in blue. The numbers of differentially expressed transcripts are also indicated.

(right panel) A volcano plot comparing transcript expression in WT with Δgcn4 cells (grown in MM. Genes induced with a fold change cut-off of ≥ 2, and p-value ≤ 10^{-4} are shown in red, and the downregulated transcripts (similar cut-off parameters) are shown in blue. The numbers of differentially expressed transcripts are also shown.

C. Correlation plots between normalised read counts of the ChIP sequencing data, from cells growin in different medium. Normalised read counts of 100000 randomly selected positions from the genome are plotted. ChIP data show enrichment/DNA binding for Gcn4 only in methionine supplemented conditions. Replicates show good correlation with a Pearson correlation coefficient, R = 0.9.
