# Supplementary material for: Genome-scale reconstruction of Gcn4/ATF4 networks driving a growth program
Source: PLoS Genet. 2020 Dec 30;16(12):e1009252. doi: 10.1371/journal.pgen.1009252 (PMC7773203; doi:10.1371/journal.pgen.1009252)
Supplement: S6 Fig — Genes differentially regulated by Gcn4 show significant overlaps between two distinct datasets, where Gcn4 was induced by different modes (Fisher exact test p < 10−10, for both activated and repressed gene comparisons). The circles on the left use data from this study (methionine induced growth program), while the circles on the right use data from a study where, Gcn4 amounts were increased using an inducible system (2 hrs post induction of Gcn4) [7]. The overlapping genes between these two datasets were enriched for amino acids biosynthesis (activated in both the datasets) and the ribosomal protein (repressed in both the datasets). Also see S6 Data. (PDF) [file pgen.1009252.s006.pdf]

A. Transcripts activated by Gcn4

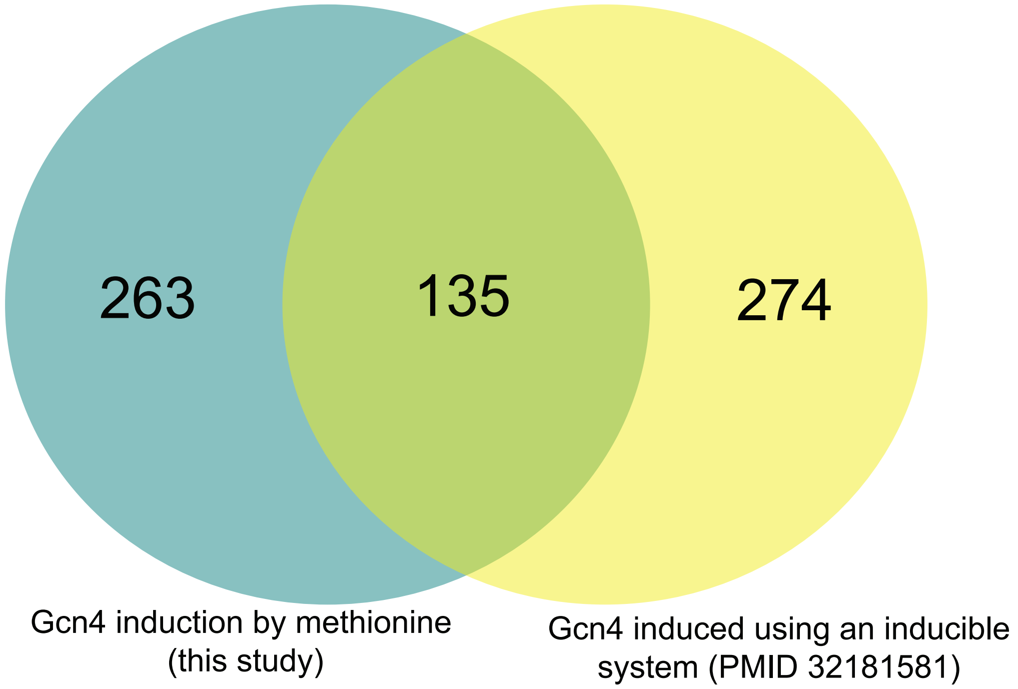

B. Transcripts repressed by Gcn4

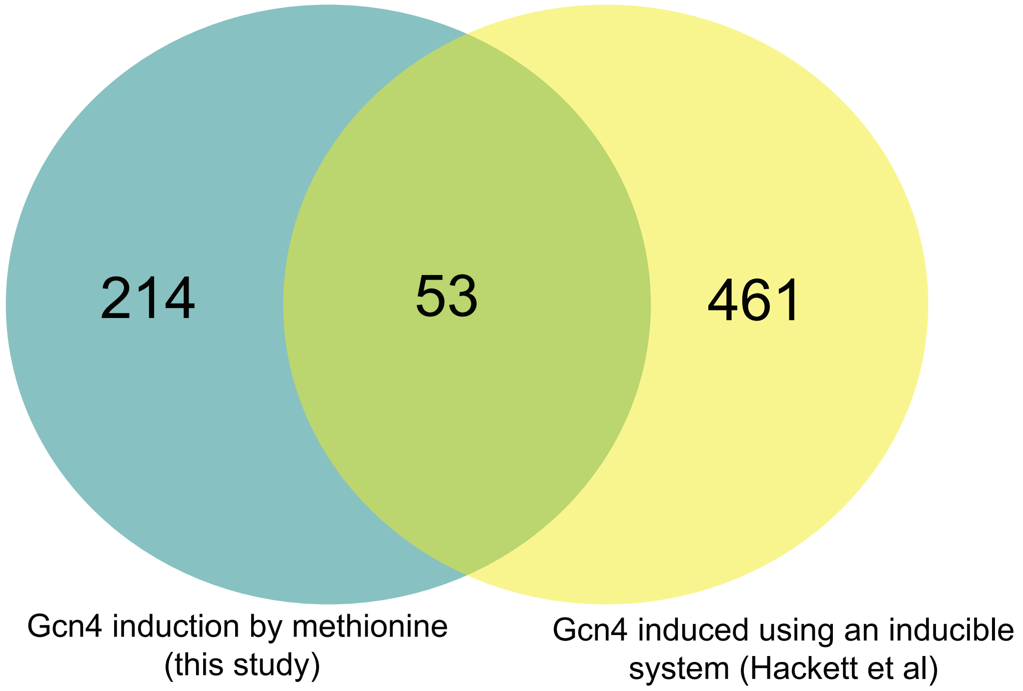

Supplementary Figure 6: Venn diagrams showing the number of differentially expressed genes that overlap, from data obtained from distinct datasets of Gcn4 levels are high.
The circles on the left use data from this study (methionine induced growth program), while the circles on the right use data from a study where, Gcn4 amounts were increased using an inducible system (2 hrs post induction of Gcn4) [7]. The overlapping genes between these two datasets were enriched for amino acids biosynthesis (activated in both the datasets) and the ribosomal protein (repressed in both the datasets). Also see Supplementary WS6.
