# Supplementary material for: Genome-scale reconstruction of Gcn4/ATF4 networks driving a growth program
Source: PLoS Genet. 2020 Dec 30;16(12):e1009252. doi: 10.1371/journal.pgen.1009252 (PMC7773203; doi:10.1371/journal.pgen.1009252)
Supplement: S7 Fig — A heatmap showing the read coverage from 1kb bases flanking the predicted translation start site (ATG) of genes, for the Gcn4 targets. The heatmap on the left shows read coverage in ChIP samples, and on the right shows coverage in mock-ChIP (control) in the MM +Met condition. The density plot in the top panel shows that most of the target genes have Gcn4 binding peaks upstream of the translation start site in ChIP samples (red) and such a pattern is absent in mock samples (blue). The deepTools package [8] was used for generating the matrix in this analysis. (PDF) [file pgen.1009252.s007.pdf]

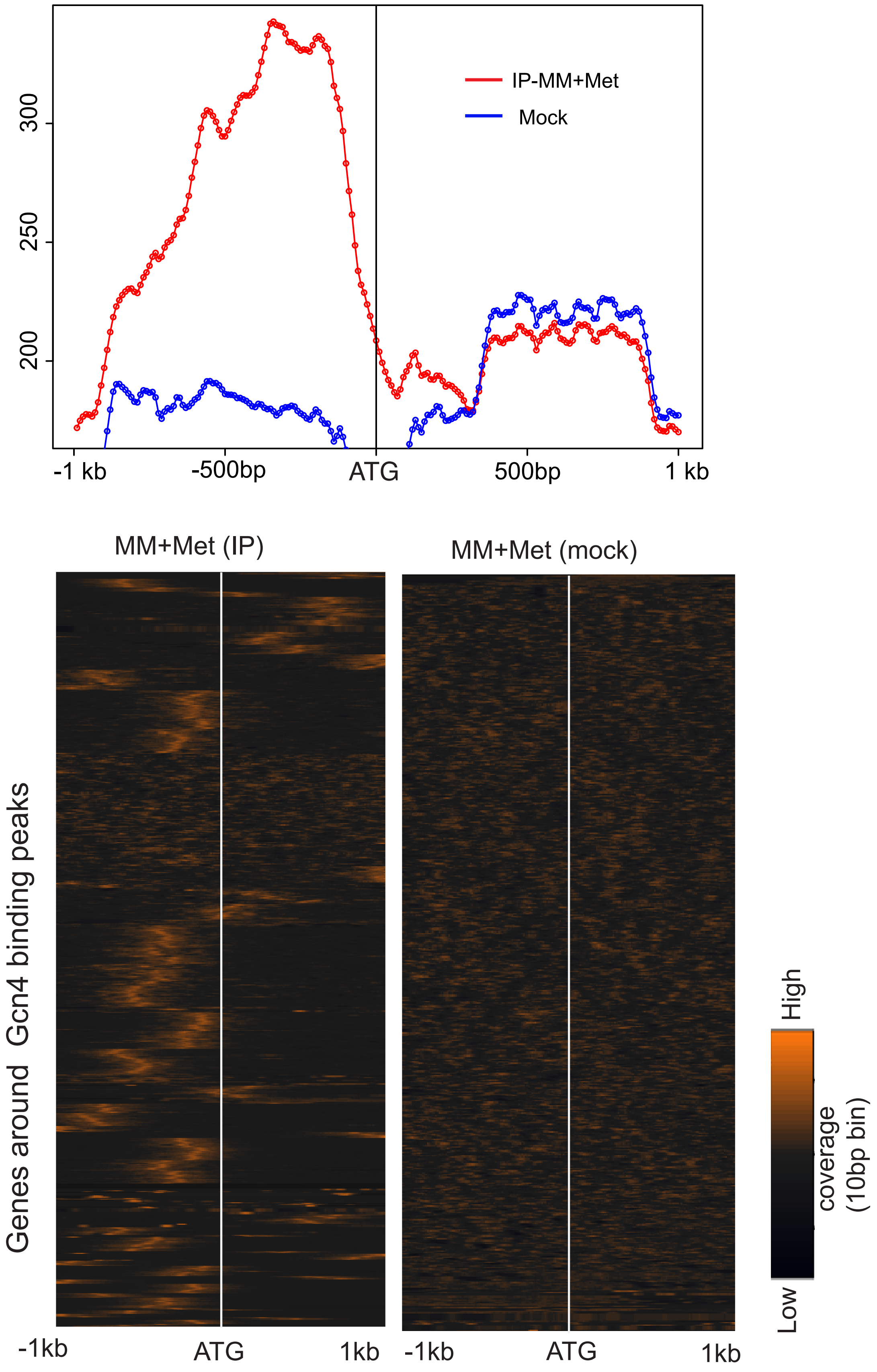

Supplementary Figure 7:
(top) Read coverage for Gcn4 binding around the translation start sites of the target genes identified by ChIP seq, in a methionine dependent growth program
(bottom) A heatmap showing the read coverage from 1kb bases flanking the predicted translation start site (ATG) of genes, for the Gcn4 targets. The heatmap on the left shows read coverage in ChIP samples, and on the right shows coverage in mock-ChIP (control) in the MM +Met condition. The density plot in the top panel shows that most of the target genes have Gcn4 binding peaks upstream of the translation start site in ChIP samples (red) and such a pattern is absent in mock samples (blue). The deepTools package [8] was used for generating the matrix in this analysis.
