# Supplementary material for: Genome-scale reconstruction of Gcn4/ATF4 networks driving a growth program
Source: PLoS Genet. 2020 Dec 30;16(12):e1009252. doi: 10.1371/journal.pgen.1009252 (PMC7773203; doi:10.1371/journal.pgen.1009252)
Supplement: S8 Fig — Gcn4 binding targets identified in the MM+Met condition were further confirmed by quantitative PCR, with the primers specific to the promoter region of the target genes. Gcn4 binding to the promoter of these targets is significantly high in MM+Met, relative to the MM. Data shown are mean±SD from 2 biological replicates. * p-value < 0.05. (PDF) [file pgen.1009252.s008.pdf]

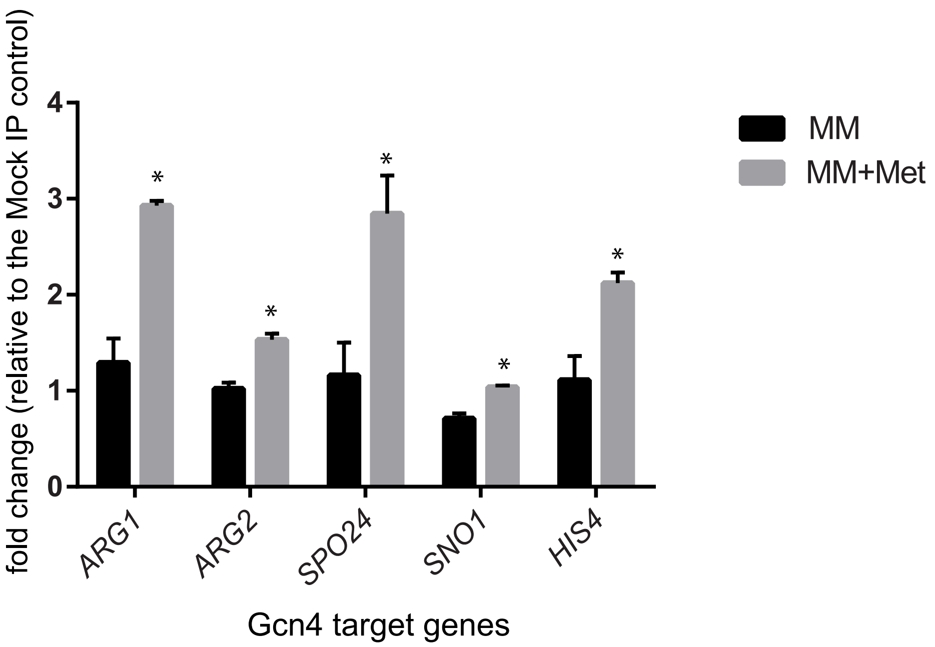

Supplementary Figure 8: ChIP qPCR validation of selected Gcn4 binding targets.
Gcn4 binding targets identified in the MM+Met condition were further confirmed by quantitative PCR, with the primers specific to the promoter region of the target genes. Gcn4 binding to the promoter of these targets is significantly high in MM+Met, relative to the MM. Data shown are mean±SD from 2 biological replicates. * p-value < 0.05.
