# Supplementary figures and images for: Genome-scale reconstruction of Gcn4/ATF4 networks driving a growth program
Source: PLoS Genet. 2020 Dec 30;16(12):e1009252. doi: 10.1371/journal.pgen.1009252 (PMC7773203; doi:10.1371/journal.pgen.1009252)

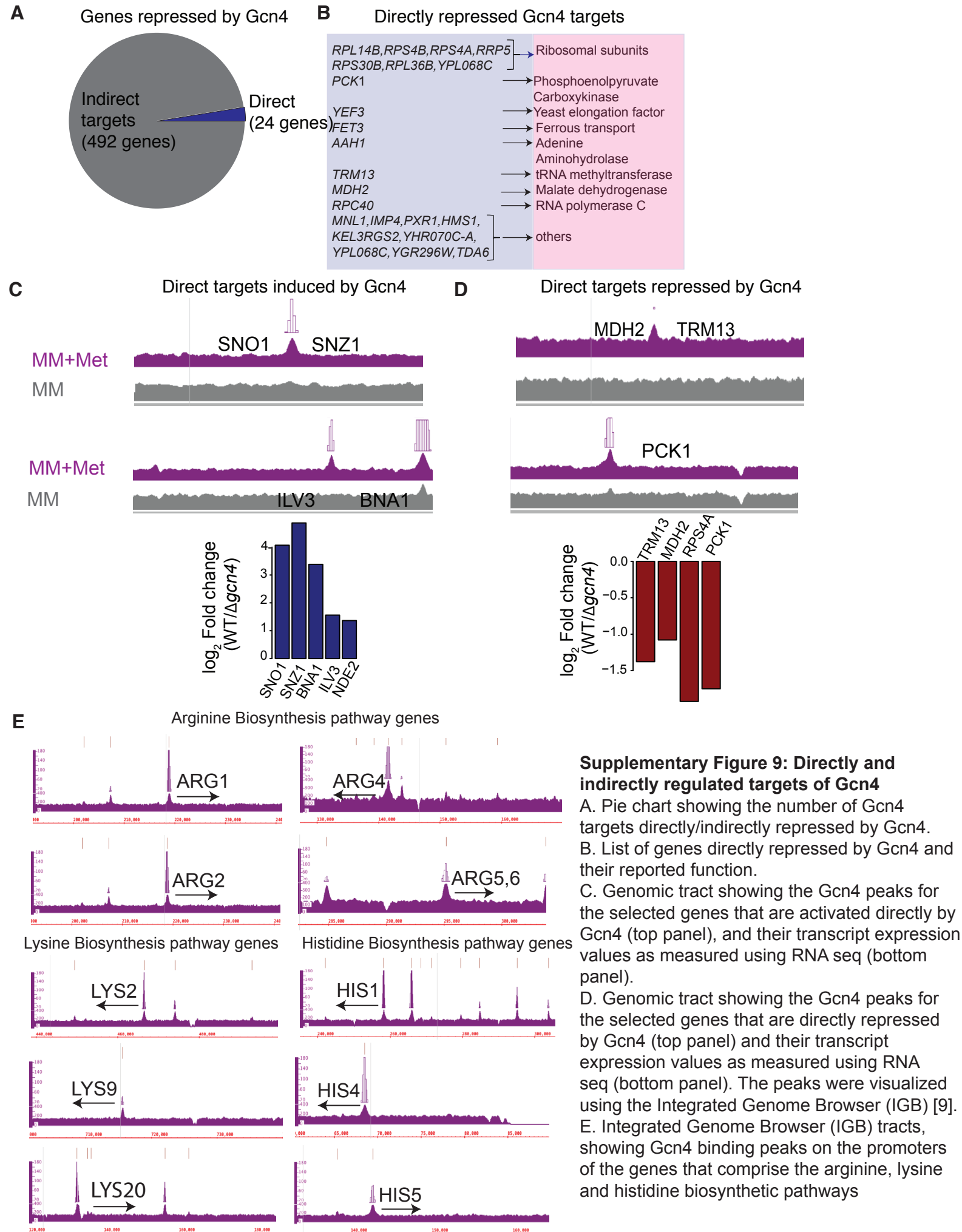

Supplement: S9 Fig — A. Pie chart showing the number of Gcn4 targets directly/indirectly repressed by Gcn4. B. List of genes directly repressed by Gcn4 and their reported function. C. Genomic tract showing the Gcn4 peaks for the selected genes that are activated directly by Gcn4 (top panel), and their transcript expression values as measured using RNA seq (bottom panel). D. Genomic tract showing the Gcn4 peaks for the selected genes that are directly repressed by Gcn4 (top panel) and their transcript expression values as measured using RNA seq (bottom panel). The peaks were visualized using the Integrated Genome Browser (IGB) [9]. E. Integrated Genome Browser (IGB) tracts, showing Gcn4 binding peaks on the promoters of the genes that comprise the arginine, lysine and histidine biosynthetic pathways. (PDF) [file pgen.1009252.s009.pdf]

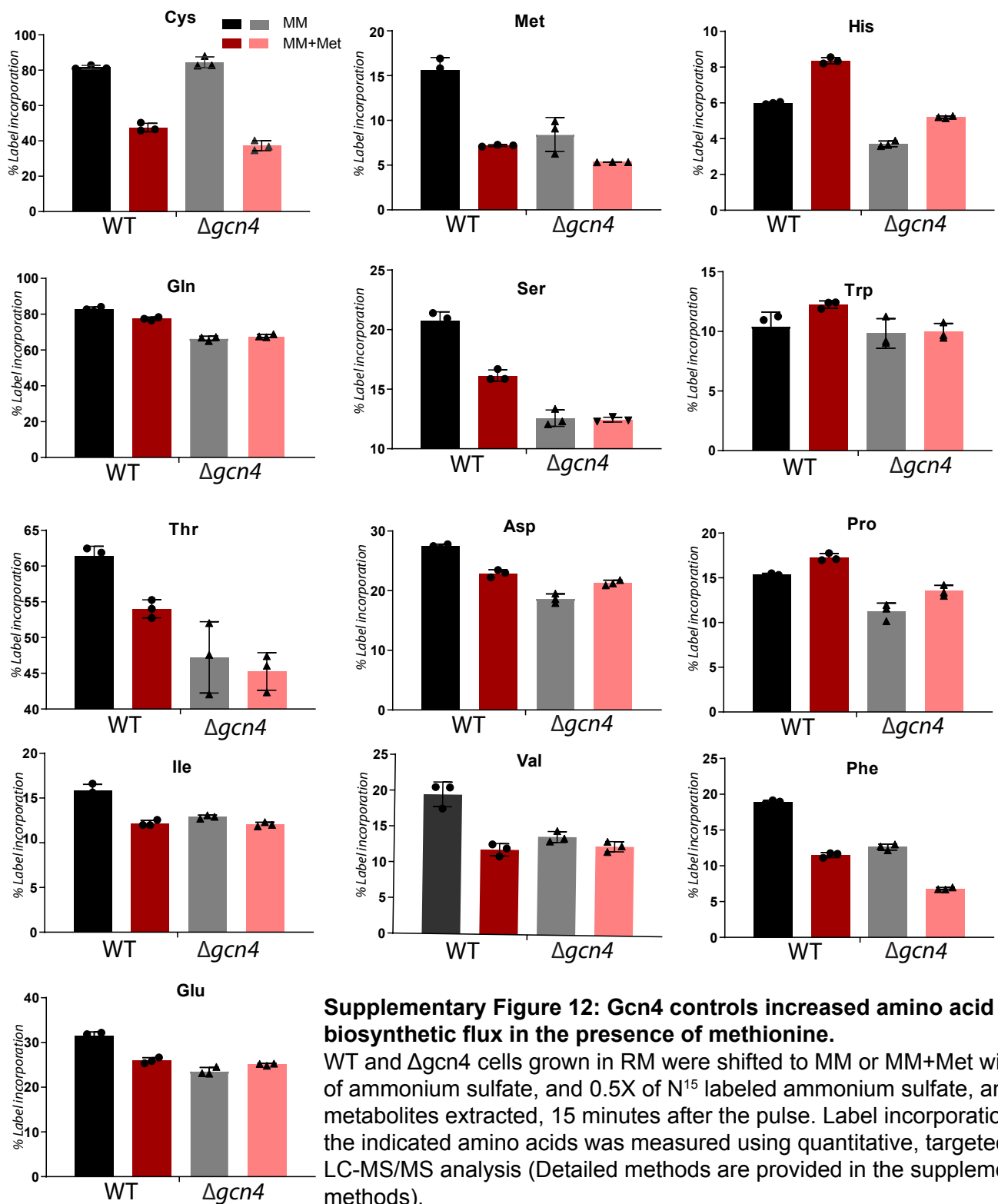

Supplement: S12 Fig — WT and Δgcn4 cells grown in RM were shifted to MM or MM+Met with 0.5X of ammonium sulfate, and 0.5X of N15 labeled ammonium sulfate, and metabolites extracted, 15 minutes after the pulse. Label incorporation into the indicated amino acids was measured using quantitative, targeted LC-MS/MS analysis (Detailed methods and raw data are provided in S1 Text and S7 Data). (PDF) [file pgen.1009252.s012.pdf]
