# Supplementary material for: Genome-scale reconstruction of Gcn4/ATF4 networks driving a growth program
Source: PLoS Genet. 2020 Dec 30;16(12):e1009252. doi: 10.1371/journal.pgen.1009252 (PMC7773203; doi:10.1371/journal.pgen.1009252)
Supplement: S10 Fig — These are compared using datasets from two different conditions (x-axis this study, y axis [10]). The expression of direct targets of Gcn4 shows a Pearson correlation r = 0.43 between the datasets. The indirect targets show a Pearson correlation r = 0.3. (PDF) [file pgen.1009252.s010.pdf]

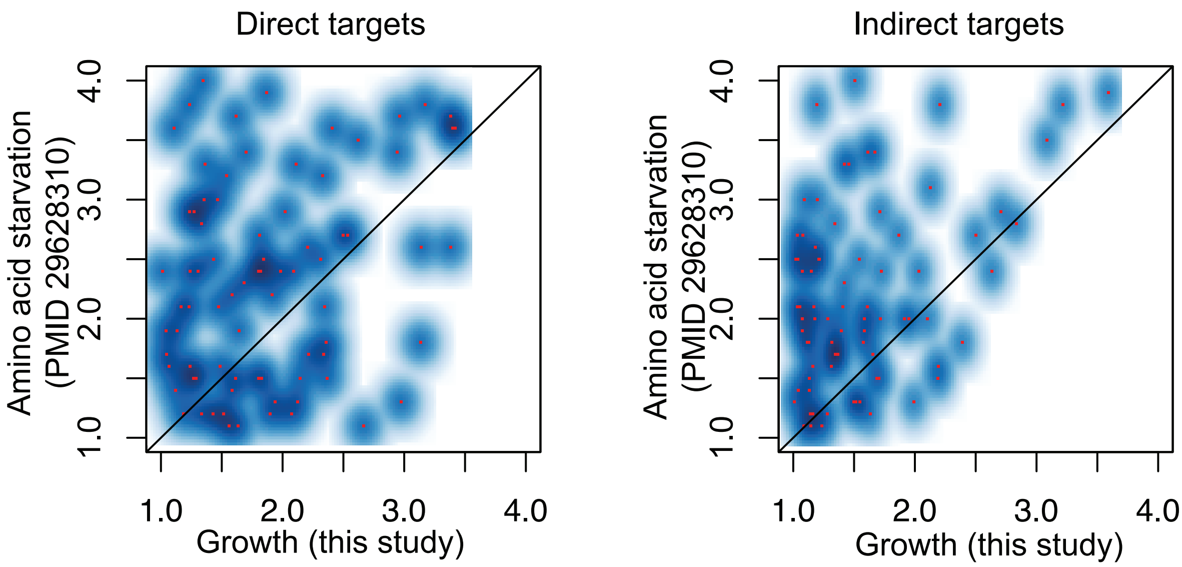

Supplementary Figure 10: Plot showing correlations for transcript expression of either direct or indirect targets of Gcn4.
These are compared using datasets from two different conditions (x-axis this study, y axis [10]). The expression of direct targets of Gcn4 shows a Pearson correlation r=0.43 between the datasets. The indirect targets show a Pearson correlation r=0.3.
