# Supplementary material for: Genome-scale reconstruction of Gcn4/ATF4 networks driving a growth program
Source: PLoS Genet. 2020 Dec 30;16(12):e1009252. doi: 10.1371/journal.pgen.1009252 (PMC7773203; doi:10.1371/journal.pgen.1009252)
Supplement: S11 Fig — For all the Gcn4 binding peaks identified in this study (x-axis), we compared the Gcn4 binding signal from two different growth conditions (y-axis left panel–from [10], right panel–from [11]) These data suggest that irrespective of the growth medium or condition, Gcn4 binding signals correlate between distinct datasets. (PDF) [file pgen.1009252.s011.pdf]

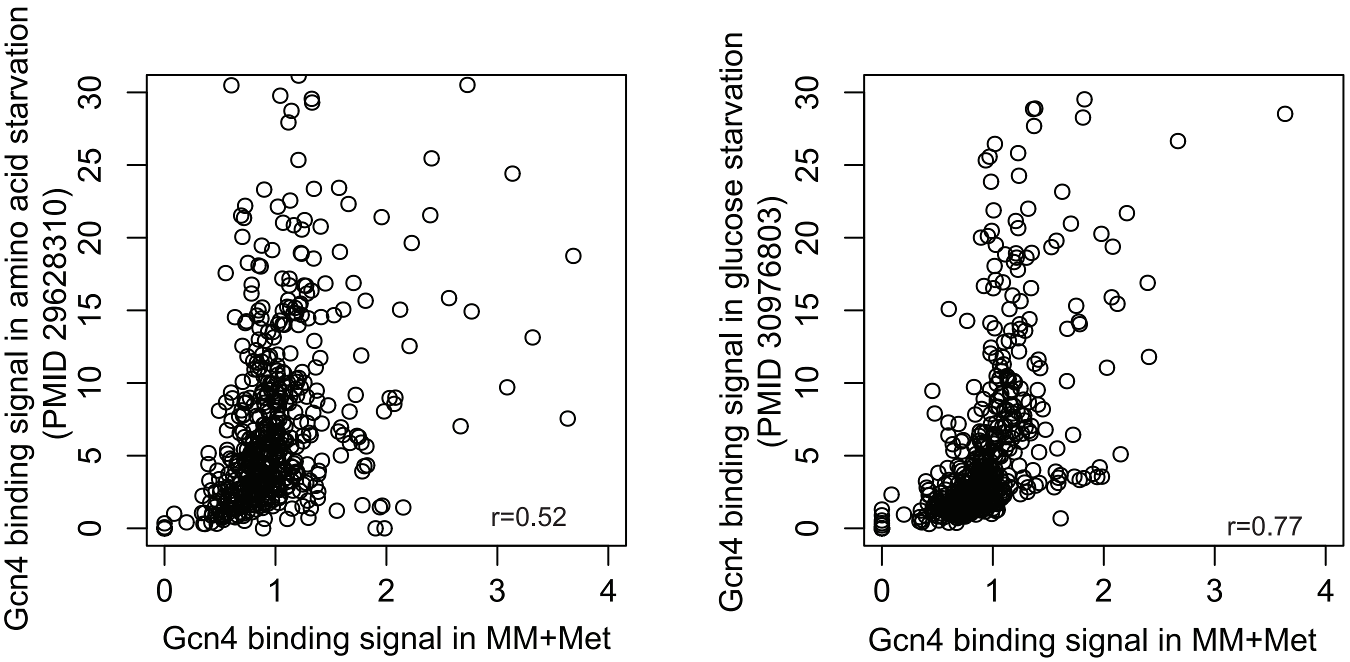

Supplementary Figure 11: Correlation of ChIP binding signals obtained from distinct growth conditions. For all the Gcn4 binding peaks identified in this study (x-axis), we compared the Gcn4 binding signal from two different growth conditions (y-axis left panel – from [10], right panel – from [11]) These data suggest that irrespective of the growth medium or condition, Gcn4 binding signals correlate between distinct datasets.
