# Supplementary material for: Genome-scale reconstruction of Gcn4/ATF4 networks driving a growth program
Source: PLoS Genet. 2020 Dec 30;16(12):e1009252. doi: 10.1371/journal.pgen.1009252 (PMC7773203; doi:10.1371/journal.pgen.1009252)
Supplement: S13 Fig — In the dataset of Gcn4 targets during amino acid starvation [10] the transcripts repressed by Gcn4 are also enriched for translation related processes, and these transcripts fall into the bin enriched for arginine and lysine codons. The left panel shows data from this study (as shown in Fig 4D), and the right panel shows a similar analysis using data from [10]. A significant enrichment of the Gcn4 targets (repressed by Gcn4) is present in bin3 (Fisher exact test: p-value < 10−10). (PDF) [file pgen.1009252.s013.pdf]

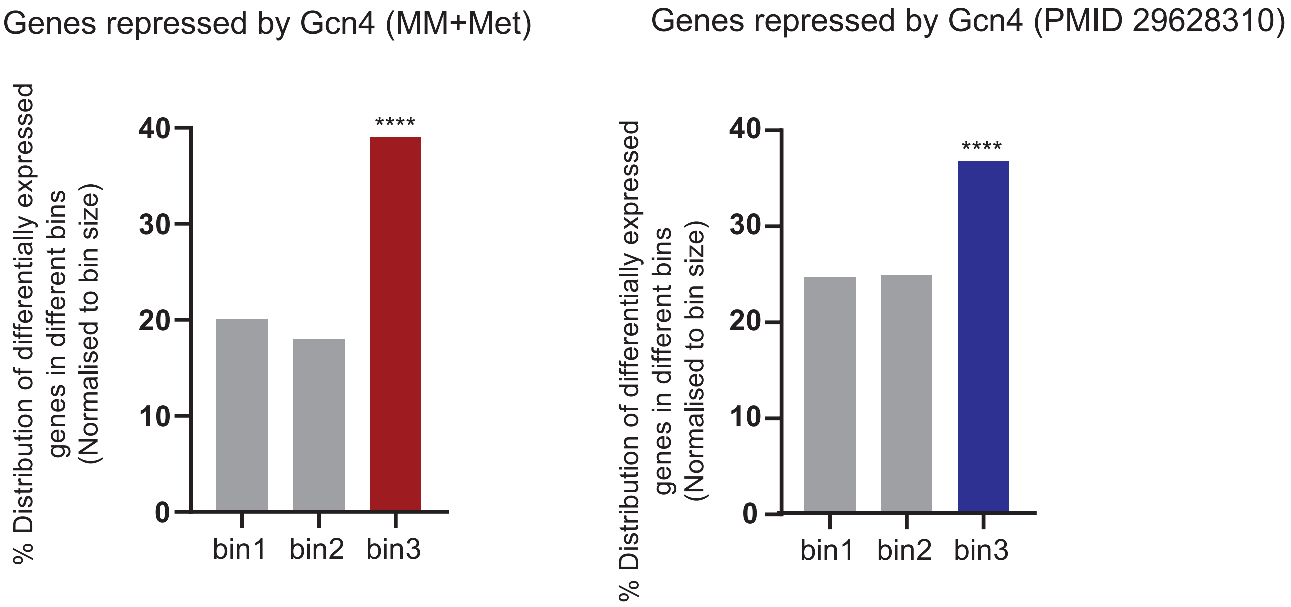

Supplementary Figure 13: Transcripts repressed by Gcn4 are enriched for arginine and lysine codons: In the dataset of Gcn4 targets during amino acid starvation [10] the transcripts repressed by Gcn4 are also enriched for translation related processes, and these transcripts fall into the bin enriched for arginine and lysine codons. The left panel shows data from this study (as shown in Figure 4D), and the right panel shows a similar analysis using data from [10]. A significant enrichment of the Gcn4 targets (repressed by Gcn4) is present in bin3.
