# Supplementary material for: Genome-scale reconstruction of Gcn4/ATF4 networks driving a growth program
Source: PLoS Genet. 2020 Dec 30;16(12):e1009252. doi: 10.1371/journal.pgen.1009252 (PMC7773203; doi:10.1371/journal.pgen.1009252)
Supplement: S14 Fig — The complete open reading frames (ORFs) of the indicated genes were cloned in place of ‘GOI-Gene of Interest’ labelled in the plasmid map. In this study, ORFs of genes RPL32, NHP2, STM1, RPS20 were cloned upstream of the luciferase sequence, and the resulting plasmids were named pSL218, pSL221, pSL224, pSL234 respectively. In this system, the expression of the GOI+luciferase is under the control of an inducible promoter, which is induced upon adding ß- estradiol, leading to gene expression and translation. The activity of luciferase enzyme (which is cloned in frame with the gene of interest (GOI)), serves as a quantitative indicator of the translation of that reporter in the given condition and genetic background. The plasmid map was created using SnapGene viewer version 3.3.4 (http://www.snapgene.com) (PDF) [file pgen.1009252.s014.pdf]

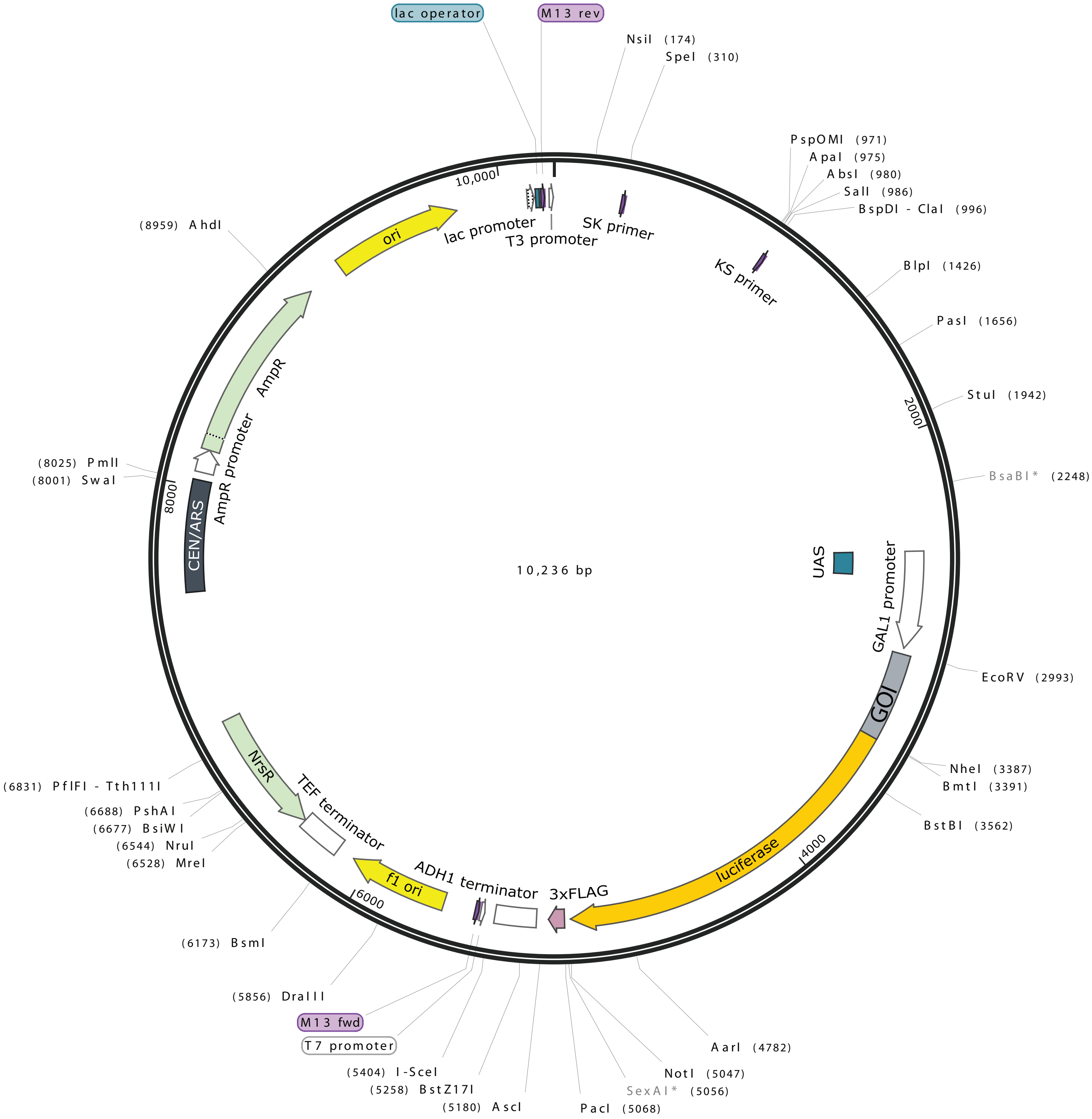

Supplementary Figure 14: Inducible luciferase-conjugated reporters to estimate expression of lysine and arginine enriched genes.

The complete open reading frames (ORFs) of the indicated genes were cloned in place of 'GOI-Gene of Interest' labelled in the plasmid map. In this study, ORFs of genes RPL32, NHP2, STM1, RPS20 were cloned upstream of the luciferase sequence, and the resulting plasmids were named pSL218, pSL221, pSL224, pSL234 respectively. In this system, the expression of the GOI+luciferase is under the control of an inducible promoter, which is induced upon adding ß- estradiol, leading to gene expression and translation. The activity of luciferase enzyme (which is cloned in frame with the gene of interest (GOI)), serves as a quantitative indicator of the translation of that reporter in the given condition and genetic background. The plasmid map was created using SnapGene viewer version 3.3.4 (http://www.snapgene.com)
