# Supplementary material for: Genome-scale reconstruction of Gcn4/ATF4 networks driving a growth program
Source: PLoS Genet. 2020 Dec 30;16(12):e1009252. doi: 10.1371/journal.pgen.1009252 (PMC7773203; doi:10.1371/journal.pgen.1009252)
Supplement: S15 Fig — Related to Fig 5D: Cells were supplemented with serine, glycine and alanine in combination (as these amino acids do not interconvert to arginine), in order to test if other amino acids could rescue the translation capacity of the Δgcn4 in MM+Met. Glycine, serine and alanine (were supplemented at 2mM each), in similar experiments to those carried out with arg/lys, and reporter activity was measured. These pooled amino acids had no effect on reporter activity in Δgcn4 cells. (PDF) [file pgen.1009252.s015.pdf]

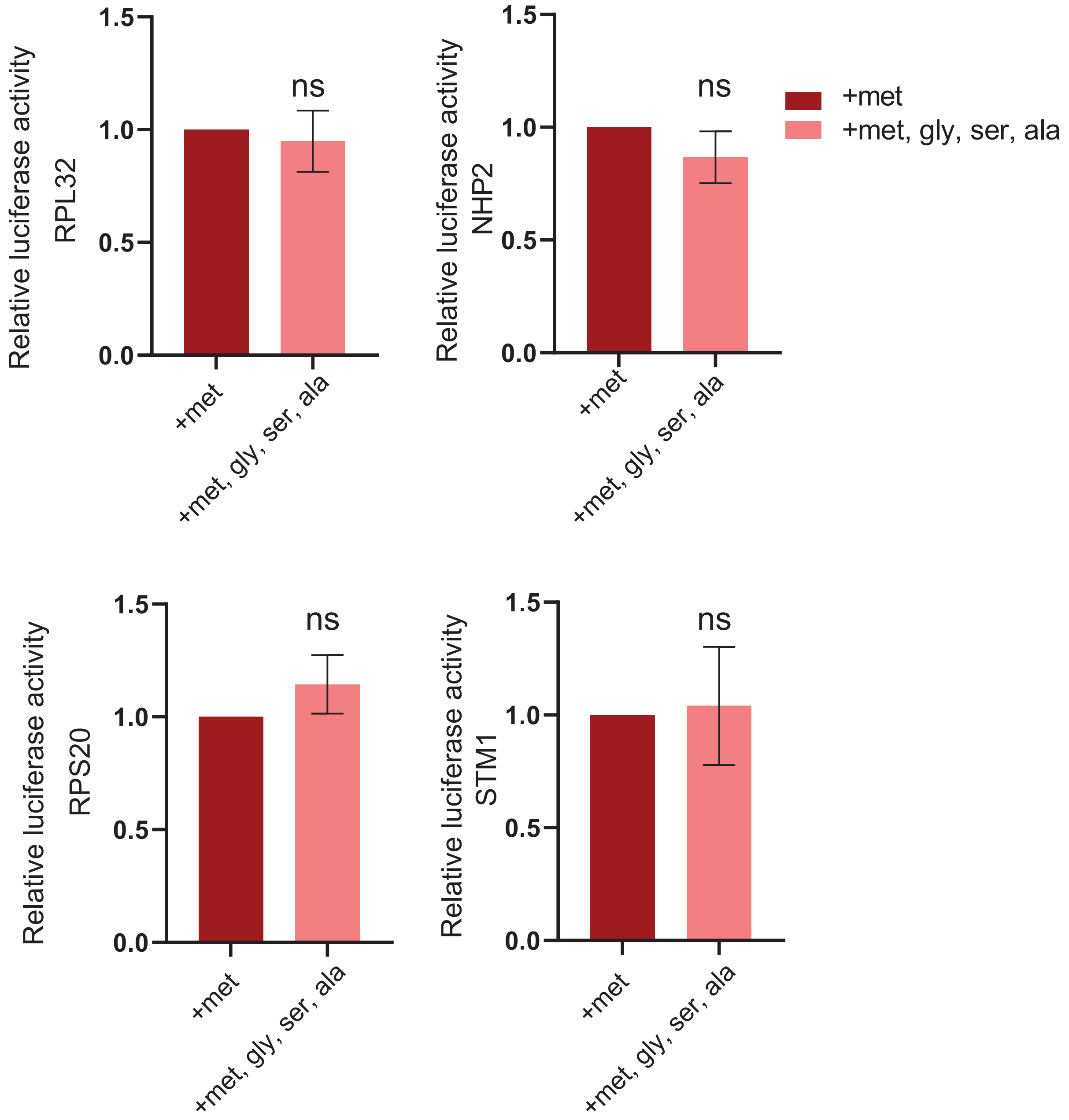

Supplementary Figure 15: Supplementing non-arginine/lysine amino acids does not increase the translation of reporter genes in Δgcn4 cells in MM+Met.

Related to Figure 5D: Cells were supplemented with serine, glycine and alanine in combination (as these amino acids do not interconvert to arginine), in order to test if other amino acids could rescue the translation capacity of the Δgcn4 in MM+Met. Glycine, serine and alanine (were supplemented at 2mM each), in similar experiments to those carried out with arg/lys, and reporter activity was measured. These pooled amino acids had no effect on reporter activity in Δgcn4 cells.
