# Supplementary material for: Genome-scale reconstruction of Gcn4/ATF4 networks driving a growth program
Source: PLoS Genet. 2020 Dec 30;16(12):e1009252. doi: 10.1371/journal.pgen.1009252 (PMC7773203; doi:10.1371/journal.pgen.1009252)
Supplement: S1 Text — (DOCX) [file pgen.1009252.s016.docx]

**S1 Text**

**Supplementary results:**

***Methionine induces a universal ‘growth program’***

Our experimental design used prototrophic yeast cells growing in synthetic minimal medium with glucose (**MM**), or this same medium supplemented with 2 mM methionine (**MM+Met**), from which RNA was extracted for deep sequencing and analysis (S1 Fig and S2A Fig) (also see Materials and methods for more details). In glucose rich medium, we found a methionine-dependent, differential expression of 969 genes (fold change > 1.5 fold). Of these differentially expressed genes, 574 genes were upregulated by methionine, and 395 genes were downregulated (S2B Fig and S1 Data). A Gene Ontology (GO)-based analysis [1] of the upregulated genes shows a significant enrichment of amino acid biosynthesis, cytoplasmic translation and ribosomal genes, mitochondrial translation, and nucleotide biosynthesis (hypergeometric test, p-value <= 0.05) (S2C Fig, and S3 Data). In contrast, the downregulated genes are involved in sulfur compound metabolic process (which is expected, since methionine is supplemented), ion transport, carbohydrate metabolism, hexose-transport (S2C Fig, S3 Data). These results show that supplementing methionine strongly induces amino acid biosynthesis, nucleotide biosynthesis and translation related genes– all of which are hallmark signatures of an anabolic program, even with glucose as a primary carbon source [2–4].

We asked how well these groupings and altered transcripts (in MM+Met) with glucose as a primary carbon source compared to earlier data from cells growing with lactate as a sole carbon source [5], to obtain a universal ‘methionine-dependent’ gene expression signature. For this, we classified genes involved in amino acid biosynthesis and nucleotide biosynthesis genes as an ‘*anabolic gene group*’, constituting 158 genes. Out of these 158 anabolic genes, ~40% of the genes are universally upregulated by methionine (63 out of 158), with a fold increase of > 1.5 fold. 67% of these induced genes overlap in glucose and lactate conditions (Fisher exact test p-value 1.4 x 10^-13^). These data show that the induction of anabolic genes by methionine is universal, regardless of the carbon source used (S2D Fig and S2 Data). Similarly, we grouped ~390 genes as ‘*translation related genes*’. Out of 390 translation related genes, ~170 genes were upregulated in glucose upon methionine addition. As with the anabolic genes considered earlier, a significant proportion of these induced genes in glucose overlapped with genes induced by methionine in cells growing in lactate (Fisher exact test p-value <10^-10^) (S2D Fig & S2 Data). We further directly tested this induction of amino acid and nucleotide biosynthesis, using a quantitative, targeted, stable-isotope pulsed LC/MS/MS based flux approach [6]. We observed a strong increase in *de novo* amino acid and nucleotide biosynthesis upon methionine supplementation, in glucose medium (S3 Fig). Thus, we find that methionine triggers a growth program, with the induction of both metabolic and ribosomal genes, even in preferred medium with glucose as a carbon source.

**Supplementary Methods:**

**Metabolite extraction and Mass spectrometry analysis:**

Cells grown in YPD were shifted to fresh minimal medium (with the appropriate carbon source as indicated), containing 0.5 X of unlabelled ammonium sulfate (0.25%) and MM+Met containing 0.5X of unlabelled ammonium sulfate (0.25%). After 1 hour of shift to minimal media, cells were pulsed with 0.25% ^15^N Ammonium Sulphate (Sigma, Cat 299286 ). Metabolites were extracted following the protocol described in [5], after 15 mins of incubation with the labelled ammonium sulfate. Briefly, ~ 10 OD_600_ of cells were quenched in 40ml of 60% Methanol at -40°C for 5 mins. The suspension was centrifuged at 1000g for 3 min at -8°C. The pellet was washed with 1ml of 60% methanol and resuspended in 1ml of 75% ethanol. It was then incubated at 80°C for 3 min and afterwards put on ice for 5 min. The suspension was centrifuged at 20000g for 1 min. 950µl of the supernatant was transferred to a fresh tube and again centrifuged at 20000g for 10 min. 300µl of the supernatant each was collected into three separate tubes (technical replicates) and put in speed-vac for drying. The dried samples were stored at –80°C till further processing. The samples were dissolved in 500ul of LC-MS grade water and run in HPLC-MS. Equal amounts of total metabolite extracts (as normalized by cell number/extraction/sample) were used in each injection, and technical replicates were used to confirm signal consistency.

A SynergiTM 4 µm Fusion-RP 80Å (150 X 4.6 mm) LC column (Phenomenex, 00F-4424-E0) was used for separation. The solvents used were 0.1% formic acid in water (Solvent A) and 0.1% formic acid in methanol (Solvent B). The gradient followed for detection was : flow rate, 0.4 ml/min; T = 0 min, 0% B; T = 3 min, 5% B; T = 10 min, 60% B; T = 11 min, 95% B; T = 14 min, 95% B; T = 15 min, 5% B; T = 16 min, 0% B; T = 21 min, stop. The Q1/Q3 values used in detection in MS/MS are given in Table 3. 10μl of sample was injected into ABSciex QTRAP 6500 with Waters Acquity UPLC system and detection was performed in positive polarity mode. Mass-Spec parameters used are as follows, declustering potential: 65V, entrance potential: 11.4V, collision cell exit potential: 12.0V, curtain gas: 45psi, ion spray voltage: 4000V, temperature: 400℃, nebulizer gas: 25psi, heater gas: 25psi. Mass-Spec data was acquired using Analyst® 1.6.2 software (Sciex). For analysis, MultiQuant^TM^ version 3.0.1 and PeakView® version 2.0 was used. Total label incorporation was determined by adding the peak area of all labels detected for a particular metabolite in a given sample. Analysed data were plotted using GraphPad Prism 6 software. Statistical significance was calculated using unpaired Student’s *t*-test**.**

**Supplementary References**

1. Raudvere U, Kolberg L, Kuzmin I, Arak T, Adler P, Peterson H, et al. g:Profiler: a web server for functional enrichment analysis and conversions of gene lists (2019 update). Nucleic Acids Res. Oxford University Press; 2019;47: W191–W198. doi:10.1093/nar/gkz369

2. Xiao L, Grove A. Coordination of Ribosomal Protein and Ribosomal RNA Gene Expression in Response to TOR Signaling. Curr Genomics. Bentham Science Publishers Ltd; 2009;10: 198–205. doi:10.2174/138920209788185261

3. Airoldi EM, Huttenhower C, Gresham D, Lu C, Caudy AA, Dunham MJ, et al. Predicting cellular growth from gene expression signatures. PLoS Comput Biol. 2009/01/02. Public Library of Science; 2009;5: e1000257–e1000257. doi:10.1371/journal.pcbi.1000257

4. Brauer MJMJ, Huttenhower C, Airoldi EMEM, Rosenstein R, Matese JCJC, Gresham D, et al. Coordination of Growth Rate, Cell Cycle, Stress Response, and Metabolic Activity in Yeast. Mol Biol Cell. 2008;19: 352–267. doi:10.1091/mbc.E07-08-0779

5. Walvekar AS, Srinivasan R, Gupta R, Laxman S. Methionine coordinates a hierarchically organized anabolic program enabling proliferation. Mol Biol Cell. American Society for Cell Biology (mboc); 2018;29: 3183–3200. doi:10.1091/mbc.E18-08-0515

6. Walvekar A, Rashida Z, Maddali H, Laxman S. A versatile LC-MS/MS approach for comprehensive, quantitative analysis of central metabolic pathways. Wellcome open Res. F1000 Research Limited; 2018;3: 122. doi:10.12688/wellcomeopenres.14832.1

7. Hackett SR, Baltz EA, Coram M, Wranik BJ, Kim G, Baker A, et al. Learning causal networks using inducible transcription factors and transcriptome-wide time series. Mol Syst Biol. John Wiley & Sons, Ltd; 2020;16: e9174. doi:10.15252/msb.20199174

8. Ramírez F, Ryan DP, Grüning B, Bhardwaj V, Kilpert F, Richter AS, et al. deepTools2: a next generation web server for deep-sequencing data analysis. Nucleic Acids Res. 2016/04/13. Oxford University Press; 2016;44: W160–W165. doi:10.1093/nar/gkw257

9. Freese NH, Norris DC, Loraine AE. Integrated genome browser: visual analytics platform for genomics. Bioinformatics. 2016/03/16. Oxford University Press; 2016;32: 2089–2095. doi:10.1093/bioinformatics/btw069

10. Rawal Y, Chereji R V, Valabhoju V, Qiu H, Ocampo J, Clark DJ, et al. Gcn4 Binding in Coding Regions Can Activate Internal and Canonical 5’ Promoters in Yeast. Mol Cell. 2018/04/05. 2018;70: 297-311.e4. doi:10.1016/j.molcel.2018.03.007

11. Holland P, Bergenholm D, Börlin CS, Liu G, Nielsen J. Predictive models of eukaryotic transcriptional regulation reveals changes in transcription factor roles and promoter usage between metabolic conditions. Nucleic Acids Res. Oxford University Press; 2019;47: 4986–5000. doi:10.1093/nar/gkz253

12. J.P. van Dijken JB, Brambillac L, Dubocd P, Francoise JM, Gancedof C, Giusepping, M.L.F.HEijnenh JJ, et al. An interlaboratory comparison of physiological and genetic properties of four Saccharomyces cerevisiae strains. Enzyme Microb Technol. 2000;26: 706–714.

13. McIsaac RS, Silverman SJ, McClean MN, Gibney PA, Macinskas J, Hickman MJ, et al. Fast-acting and nearly gratuitous induction of gene expression and protein depletion in Saccharomyces cerevisiae. Mol Biol Cell. 2011/09/30. The American Society for Cell Biology; 2011;22: 4447–4459. doi:10.1091/mbc.E11-05-0466
