# Supplementary material for: Genome-scale reconstruction of Gcn4/ATF4 networks driving a growth program
Source: PLoS Genet. 2020 Dec 30;16(12):e1009252. doi: 10.1371/journal.pgen.1009252 (PMC7773203; doi:10.1371/journal.pgen.1009252)
Supplement: S1 Table — (DOCX) [file pgen.1009252.s017.docx]

**S1 Table: List of strains**

| Strains | Genotype | Reference |
| --- | --- | --- |
| WT (CEN.PK) | Mat a | S1 Text Reference [12] |
| Gcn4-HA | MAT a, *GCN4-HA-KanMX* | S1 Text Reference [5] |
| Gcn4-FLAG | MAT a, *GCN4-FLAG-KanMX* | This Study |
| *gcn4Δ::NAT* | MAT a, *gcn4Δ::NAT* | S1 Text Reference [5] |
| *gcn4Δ::KanMX* | MAT a, *gcn4Δ::KanMX* | This Study |
