# Supplementary material for: Genome-scale reconstruction of Gcn4/ATF4 networks driving a growth program
Source: PLoS Genet. 2020 Dec 30;16(12):e1009252. doi: 10.1371/journal.pgen.1009252 (PMC7773203; doi:10.1371/journal.pgen.1009252)
Supplement: S2 Table — (DOCX) [file pgen.1009252.s018.docx]

**S2 Table : List of plasmids**

| Plasmid | Promoter and Reporter | Reference |
| --- | --- | --- |
| pGL3-basic |  | Addgene |
| pGEV-FLAG | GEV- estradiol inducible promoter | S1 Text Reference [13] |
| pSL207 (modified version of pGEV-FLAG) | GEV- estradiol inducible promoter | This study |
| pSL217 | *pGEV-Luciferase::NAT* | This study |
| pSL218 | *pGEV-RPL32-Luciferase::NAT* | This study |
| pSL221 | *pGEV-NHP2-Luciferase::NAT* | This study |
| pSL224 | *pGEV-STM1-Luciferase::NAT* | This study |
| pSL234 | *pGEV-RPS20-Luciferase::NAT* | This study |
