# Supplementary material for: Genome-scale reconstruction of Gcn4/ATF4 networks driving a growth program
Source: PLoS Genet. 2020 Dec 30;16(12):e1009252. doi: 10.1371/journal.pgen.1009252 (PMC7773203; doi:10.1371/journal.pgen.1009252)
Supplement: S3 Table — (DOCX) [file pgen.1009252.s019.docx]

**S3 Table:** List of Q1/Q3 for 15N-labelled metabolites detection by LC-MS/MS. The numbers after the underscore in the metabolite name indicates increase in mass eg: In 15N_Arginine_2, 2 indicates +2 increase in mass, corresponding to +2 label.

| S. no. | Metabolite | Q1/Q3 | CE | RT |
| --- | --- | --- | --- | --- |
| 1 | Arginine | 175.2/60 | 14 | 4.21 |
| 2 | 15N_Arginine_1 | 176.2/61 | 14 | 4.21 |
| 3 | 15N_Arginine_2 | 177.2/61 | 14 | 4.21 |
| 4 | 15N_Arginine_3 | 178.2/61 | 14 | 4.21 |
| 5 | 15N_Arginine_4 | 179.2/61 | 14 | 4.21 |
| 6 | Lysine | 147/84.1 | 16 | 4.07 |
| 7 | 15N_Lysine_1 | 148/85.1 | 16 | 4.07 |
| 8 | 15N_Lysine_2 | 149/85.1 | 16 | 4.07 |
