# Supplementary material for: Genome-scale reconstruction of Gcn4/ATF4 networks driving a growth program
Source: PLoS Genet. 2020 Dec 30;16(12):e1009252. doi: 10.1371/journal.pgen.1009252 (PMC7773203; doi:10.1371/journal.pgen.1009252)
Supplement: Attachment — Submitted filename: Response_PLoSgen.docx [file pgen.1009252.s027.docx]

Dear editors,

We thank the reviewers for a critical, thorough revision of the manuscript. We now enclose a revision, where we have addressed all reviewer concerns as best as possible. For your convenience, we have also uploaded a marked version of the manuscript, with all changes made indicated with yellow highlighting.

A detailed, point by point response to each reviewer comment is provided later in this letter. Immediately below, we have summarized responses to the points highlighted by the editors. We hope our response satisfies all the reviewer queries.

best regards,

(Sunil Laxman)

Editor summary:

1. Reviewer #2 notes that only 2 replicates were used for the metabolomics experiments - The Metabolomics Society recommends a higher number of replicates and suggests that 3 is the bare minimum that should be considered for publication. In order to ensure rigor and reproducibility we will require this critique be addressed with additional experimentation.

\#### We have now repeated these metabolic flux experiments with three biological replicates. The data and conclusions are largely unchanged. We hope this is satisfactory.

Minor point: We do note that the experiments shown were not steady-state measurements (where greater n’s are the norm), but stable-isotope based flux experiments, which were independently performed. While it is a norm for n=3 for steady state measurements, for the more challenging (and accurate) flux experiments, two independent replicates are routinely used, especially when the changes observed are very large.

2. A common theme in the reviewer comments is the need to tone-down the text. Even the most complimentary reviewers/editors felt the use of exaggerative language was hurting rather than helping the manuscript.

\#### We apologize for this. We have thoroughly revised the manuscript, and toned down all statements. We have also thoroughly revised the text to make it more succinct, and precise.

3. Some of the reviewers noted English usage errors - please note that should your manuscript be accepted PLOS does not offer proof reading services so it is critical that these issues be corrected during the revision process. Some authors find it helpful to have the manuscript proof read by colleagues while others uses professional editing services. Similarly one reviewer noted that the references needed serious editing - please be sure this is addressed.

\##### We have very carefully proofed this manuscript, and removed errors. We also apologize for the issue with the inserted reference (for which we blame the reference manager integrating with Word). We have manually inspected and corrected the issues with the references.

4. Two of the reviewers commented on data availability. Please note that PLOS Genetics requires all supporting data to either be presented in the paper, in the supplementary material, or deposited in a stable public database. This requirement is non-negotiable and must be addressed.

\##### We have ensured that all data is fully available. For the mass spectrometry data, we have now provided extensive details (including a detailed supplementary section with methodology details, and raw MS/MS data in Supplementary WS7) and clarified necessary points, as requested by the reviewer.

Our detailed responses to reviewer comments is below:

Reviewer 1:

We thank reviewer 1 for a very clear review.

Figure 5D: The authors need to test if other amino acid supplements beyond arg/lys increase reporter output (it would also be good to test arg and lys separately). If they are truly privileged, it might even be worth adding this result to the title of the paper.

\#### This is a fair point to make. Some amino acids can interconvert to arginine/lysine (eg glutamine). Therefore, we specifically addressed this, by supplementing cells with other amino acids that do not as easily interconvert to arginine, but also (in distinct contexts) have been shown to help ‘growth’. Specifically, we investigated if other amino acids could rescue the translation capacity of the *Δgcn4*. We supplemented a mixture of the amino acids glycine, serine and alanine (all at 2mM each), in very similar experiments to those carried out with arg/lys, and measured reporter activity. These amino acids *combined* had no effect on reporter activity in *Δgcn4* cells, and did not rescue the translation capacity. This further supports our claim that the arginine and lysine supply is important to rescue the translation capacity of the cells lacking Gcn4.

We include this control as a supplementary figure 15 (shown below), and also make the appropriate changes in text (438-443).

*“To evaluate the specific requirement of arginine and lysine, we also included other amino acid controls. Here, three distinct amino acids (serine, glycine and alanine) which cannot easily interconvert to arginine or lysine, were supplemented as a pooled mixture along with methionine (all at 2mM each), and the luciferase reporter activity was measured These amino acids combined had no effect on reporter activity in Δgcn4 cells, and did not increase the translation of these reporter proteins (Supplementary Figure 15). “*

Page 3, Line 111: Is methionine the only amino acid that induces a "growth signature" expression program?

####We have not tested the extent of gene expression changes upon supplementing each individual amino acid. However, in our earlier study (PMID 30354837), where we first identified the growth program induced by methionine, we had extensively investigated the combined role of all other amino acids (where met and cys were excluded). In that study, we found that the addition of *all* other non sulfur amino acids to the cells growing in amino acid limited conditions did not induce a ‘growth’ transcriptional signature. The transcriptome profile of the cells supplemented with all other non-sulfur amino acids was comparable to that of the signature in MM (PMID 30354837).

We have added clarifying lines in the text (119-120).

We have also added an additional piece of data, in the new Figure 1A, where we show WT and ∆gcn4 growth in MM, or MM+Met. The increased growth in +Met is very clearly seen, as is the severe growth limitation in ∆gcn4 cells in these conditions.
Page 3, Line 127: Can the overlap between the response on lactate/glucose be further quantified? "overlapped well" is a little too qualitative.

####We have currently included a heat map that compares the response in lactate and glucose, for the fold changes in expression (shown as supplementary Figure 2D.) Below are the statistics for the same.

We now edit the main text (lines 129-151), and add the relevant statistics, as indicated below:

“*Specifically, in order to compare the methionine dependent response across carbon sources, we first classified transcripts involved in amino acid biosynthesis and nucleotide biosynthesis genes as the ‘anabolic gene group’ (constituting 158 genes). Out of these 158 genes, ~40% of the genes are upregulated in MM_gluc_+Met (63 out of 158), with a ≥1.5 fold increase. 67% of these genes induced in MM_gluc_+Met overlap with genes induced in MM_lactate_+Met (Fisher exact test p-value < 10^-10^). This indicates that the induction of transcripts representing anabolic genes by methionine is universal, regardless of the carbon source (Supplementary Figure 2D). Similarly, we grouped ~ 750 transcripts representing ‘translation related genes’. Out of 750 translation related transcripts, ~ 175 were upregulated in MM_gluc_+Met. Similar to the anabolic genes considered earlier, the transcripts induced by methionine (with glucose as a carbon source) significantly overlapped with transcripts induced by methionine (with lactate as a carbon source) (Fisher exact test p-value < 10^-10^) (Supplementary Figure 2D). These results collectively indicate that the transcriptional response to methionine retains all the hallmarks of an anabolic growth program even when glucose is used as a carbon source. This includes the induction of appropriate metabolic genes (particularly all amino acid biosynthesis, nucleotide biosynthesis and transamination reaction related genes), as well as cytoplasmic translation related genes (Supplementary Figure 2, Supplementary WS2, Supplementary WS3). This induction of the translation machinery along with amino acid and nucleotide synthesis genes are classic signatures of an anabolic program [22,33,34]. These transcriptional changes functionally result in an appropriate metabolic state switch (increased de novo amino acid and nucleotide synthesis), as determined using a quantitative, targeted, stable-isotope pulsed LC/MS/MS based flux approach (Supplementary Figure 3). We therefore use this system (MM+Met) to address universal principles of cell growth regulation, and address the role of Gcn4 in this growth program. (Supplementary Figures 2, 3, 4, Figure 1A, Supplementary WS 2, 3)*

A recent publication from Hackett et al includes gene expression profiling of cells following induction of GCN4 (<https://www.embopress.org/doi/10.15252/msb.20199174>) -- it would be interesting to see if there is a signature of ribosomal gene repression in those data. These data have a benefit that they induce a single TF without other chemical perturbations, so the direct/immediate effects of GCN4 activation can be gleaned. It would also be interesting to compare the GCN4 induction data to your ChIP results.

####This is an excellent reference pointed out by the reviewer (which we had inadvertently missed). Comparing our datasets with the Hackett dataset, we find that the overlapping genes activated by Gcn4 in both these datasets are enriched for the aminoacid biosynthetic processes, and the overlapping genes repressed by Gcn4 are enriched for cytoplasmic translation. We compared the microarray data from Hackett et al, where Gcn4 translation was induced under an inducible promoter, and the Gcn4 role in gene expression was measured at several time points. We used the data after 2hrs of Gcn4 induction, with our gene expression data. In Hackett et al, ~267 genes were repressed, with the fold change of at least 2 fold. Of this ~ 20% of the genes overlap with the Gcn4 targets in growth conditions (Fisher exact test p-value < 10^-10^ ) and of the 410 genes activated in Hacketts et al, 33 % overlap with the genes activated by Gcn4 when cells are in a growth state (this study) (Fisher exact test p-value < 10^-10^). The overlapping genes between these two datasets were enriched for aminoacid biosynthesis (activated in both the datasets) and the ribosomal protein (repressed in both the datasets)

We have shown this overlap as Supplementary figure 6 and the Supplementary WS6). The following lines have been added to the main text (lines 210-216):

*“We independently carried out a similar analysis using data from a distinct study where Gcn4 expression was increased using an inducible promoter (no chemicals were used to induce starvation) [42]. This comparison also showed a significant overlap between the transcripts regulated by Gcn4 under different modes of induction (Supplementary Figure 6). A GO grouping of the genes which overlap between the growth and the starvation condition suggests a well conserved role of Gcn4 in inducing amino acid biosynthetic genes, and in repressing translation related genes (Supplementary WS6).”*

The new figure (shown below) and legend provide more information.

**Reviewer #2:** The manuscript (MS ID: PGENETICS-D-20-00597) entitled 'Genome-scale reconstruction of Gcn4/ATF4 networks driving a growth program', which has been submitted to PLOS Genetics by Srinivisan et al., illustrate how growth and starvation outcomes are controlled using the same Gcn4 transcriptional outputs. Relevant and timely study. However, the following comments / suggestions/ advice are provided to help the authors improve on the currently submitted version of the manuscript only.

\#### Thank you for a clear and concise review.

Critique and concerns for improvement:
A lot is left for imagination : “using a targeted LC/MS/MS based approach [56] to measure amino acid synthesis flux, based on stable-isotope incorporation.”

\###### Thank you for pointing this out. These data and explanations are now provided in extensive detail as an entire section of supplementary methods, on “Metabolite extractions and mass spectrometry methods”, with all fragmentation patterns and MS parameters included in Supplementary Table 3, and raw data in Supplementary WS7. We have also included a little more clarifying text in the main results (lines 315-325).

Metabolomics raw data can be shared/ provided in popular data repositories dedicated for metabolomics data.

\###### We have now included raw metabolic flux data as a supplementary worksheet (WS7). Since these were very targeted experiments for amino acid biosynthetic flux, the data generated is relatively small so need not be deposited in a repository.

L666: Which R-packages were used? Name them for individual tasks. Also, for FAIR practices provide codes as supplementary materials.

\###### This is now indicated in full detail, in the methods section of the manuscript, and we explain how the pipelines were run.

Quoting from the revised manuscript (methods section):

*“The EdgeR package [68] was used for normalization and differential gene expression analysis. Differentially expressed genes with a fold change above 1.5 or 2 fold, with a stringent p-value cutoff of <= 0.0001 were considered for further analysis. Normalized read counts was calculated for every sample as described earlier [15]. Normalized read counts between the replicates are well correlated with the Pearson correlation coefficient (R) is more than 0.9 (Supplementary figure 1). GO analysis of the differentially expressed genes were carried out using g:Profiler [69].”*

And, for the ChIP-seq analysis:

*“ChIP sequencing reads were mapped to the S. cerevisiae S288C genome downloaded from SGD. The reads with mapping quality < 20 were discarded, and the remaining reads were used for further analysis. The number of reads mapped to every 100bp non-overlapping bins were calculated using ‘exomedepth’ function of R-package GenomicRanges [71]. Read counts were normalized by dividing the number of reads falling within each bin by the total number of reads fall within the range of µ±x, where, µ=mode of the distribution of read counts of each bin, x = median absolute deviation of all the bins that has a number of reads that are less than the mean of the distribution. Subsequently, the regions with normalized read counts of above 2 were considered for further analysis. The binding regions separated by < 200bp were merged to give a single peak. Peaks which are conserved in both the replicates with the overlap of at least 50bp were considered as bona fide binding regions of Gcn4. Genes which are encoded around 750 bp on both sides of the peaks are listed in the Supplementary WS4.”*

***Peak feature annotation and motif analysis***

*Genomic features of the peaks were identified using the annotatePeak.pl function of the HOMER tool [44]. For motif analysis, nucleotide sequences corresponding to the peak intervals were extracted from the genome and motif identification was performed using ‘meme’ function of MEME-suite [45].”*

Since these were standard R-package pipelines that were executed, no additional code needs to be deposited.

L671: Remove the word “extensive”…

\###### Done. We have also carefully checked for other adjective usage, and removed most of them.

L630: instead of citing references: [20,56] provide details as supplementary materials or leave the protocols as Protocols.io and then cite them in this manuscript.

\##### We now have added an entire extensive section in the supplementary materials, for the metabolic flux experiments and all other methods, explaining in detail how the experiments were done. We have also included raw data (Supplementary WS7) for the flux/label incorporation experiments.

Major concern: L550: “platform. For every experimental condition, data were obtained from two biological replicates.” N=2 is not standard and reproducible. How would the authors defend their work in terms of effect size and statistical robustness/ considerations ?

\##### We have now repeated these metabolic flux experiments with three biological replicates. The data and conclusions are largely unchanged. We hope this is satisfactory.

Minor point: We do note that the experiments shown were not steady-state measurements (where greater n’s are the norm), but stable-isotope based flux experiments, which were independently performed. While it is indeed a norm for n=3 for steady state measurements, for the more challenging (and accurate) stable-isotope based flux experiments, two independent replicates are routinely used. This is especially so when the changes observed between the two samples that are being compared are very large, and replicates are very similar (and reproducible).

Discussion is too short.

\### We have substantially worked on the discussion, to make the main take home messages clearer, and clarified important points. We hope this discussion is more satisfactory.

Results sections are “too long” instead laced with too much citations, discussions and interpretations.

\#### We have thoroughly edited the entire results section, to state only the results and conclusions, followed by a summarizing line. We have removed excessive interpretations, and moved “discussion elements” to the discussion section, where they belong. This will be very apparent in the “marked” version of the manuscript.

Materials and methods are very well detailed.
Unsure why 2 sets of references have been added to the document and needs to be amended.

\#### We apologize for this inadverted error due to a reference manager. We have manually edited references to ensure that errors are removed.

Figure 1B: Use “transcripts” instead of genes.

\#### Done.

Major language related improvement needed. Present version has some common, punctuation, tense, grammatical, typological sentence framing/ phrase construction issues which should be taken care by the authors/ collaborators. Many loosely constructed sentences floating around.

\#### We have now carefully proofed the manuscript, and ensured consistency in tense, voice and grammar. We have also fixed typographical errors.

Please change and improve upon the conclusion to provide very specific and key take-home messages to the readers and should be only based on the findings and not extrapolations/ future aspects. Currently too long.

\#### We have revised and edited the discussion to make it tighter, and clearly provide take-home messages. We have minimized unnecessary extrapolations in the text. This can be seen in the revised discussion (and the document with marked changes clearly illustrates these points).

References are NOT OK and poorly organized in terms of formats. Follow the journal format typically as instructed to the authors. Check all the references one by one manually, so that everything cited in the text are also listed in the list of references.

\#### We apologize for this inadverted error due to a reference manager. We have manually edited references to ensure that errors are removed, and the formatting is consistent (and follows journal requirements).

If at all a revised version is submitted, then please make all changes in differently colored fonts (red/blue) or highlighted background (yellow) so that it would save the reviewer's time in finding the changes with lesser efforts and time.

Please also incorporate all the answers/ discussions/ points raised by this reviewer into the manuscript rather than just explaining to this reviewer- in order to make the manuscript stronger than it is now.

\#### Please also see the revised document with all changes made being “marked” (either with highlighted text, or cross-outs), to note the extensive changes.

**Reviewer #3:** The authors elegantly characterise the role of GCN4 in growth conditions. They show that it indeed regulates a similar set of genes as during starvation (for which the gene is much better known). They propose an interesting indirect regulatory mechanism whereby GCN4 regulates the production of specific amino acids (arginine and lysine), which are specifically enriched in a set of genes translation related genes. They thus propose that GCN4, through regulation of its direct targets, also indirectly regulates the translation of translation genes.

Overall, the study is well carried out, follows a logic flow and reveals a conceptually interesting regulatory mechanism. Throughout the manuscript - in particularly in the beginning - there are several overinterpretations of their results, so the authors should go over the text again and make sure the conclusions they draw at each stage are indeed supported by the data they present. The study is very nice and the results very solid, thus making those overstatements just hurts the credibility of the latter parts.

\#### Thank you for a very clear and constructive review.

Specific comments:

- please remove all the "striking", "strikingly" etc. There is also no need to keep repeating that the starvation program has been studied before i.e. all the "conventional" in front of starvation program can be removed, it suffices to state the in the intro and then just use it as comparison.
\#### We are sorry about that. We have edited the text (and minimized the use of adjectives like striking/ly, and reduced the excessive text related to starvation programs. This has been done throughout the manuscript.

- the beginning of the study feels like the authors are building a strawman (GCN4 has only been studied in stress, so we are doing something else). I would find it conceptually more attractive to build the story about context-dependency of GCN4 (which is essentially what the authors study). That is of course only a suggestion.

\#### This is a fair point, and we did not intend to overstate a strawman, only to demolish it. We have now revised the text, to make it more nuanced (and indicate the context dependent roles of Gcn4). The thoroughly revised text (marked) should indicate this clearly.

The most relevant regions are lines 77-93 (introduction), and lines 155-162 (results).

- Fig 1: instead of the simple overlaps in the venn diagram it would be more informative to show how the fold-changes correlate. Is it in principle the same gene expression program that gets activated?

###Yes, this is correct (explanation and changes below).

That is very difficult to judge from the Venn diagram.

We understand the concern of the reviewer. We now include a figure (new Figure 1E, shown above) showing the gene expression values as a heatmap as well, to enable the reader to visualise the data better. This is a bit subjective (and some students preferred the venn diagrams, for simplicity), so now we have both formats presented.

The heatmap shows transcript fold changes pre and post the induction of Gcn4, from different conditions. The expression trends between the growth and starvation conditions are similar. The fold changes seen due to pharmacological inhibition of amino acid biosynthesis (by SM) is slightly higher than the growth condition with methionine. This could be explained by the differences in Gcn4 protein levels, due to entirely distinct modes of induction.

- similarly for the direct vs indirect targets, do the direct target genes (i.e. those with a GCN4 motif or ChIP-seq peak) correlate better between starvation and growth, than the indirect ones?

The expression of direct targets of Gcn4 shows a better correlation between the datasets from distinct conditions (Pearson correlation r=0.43), as compared to the indirect targets (r = 0.3). There are slight differences at the fold change at which these genes are induced or repressed. This data is shown in a new Supplementary figure 10 (which is included below for convenience).

- Fig 2B: what are the genes displayed, are these only those that are differentially expressed in GCN4? Should be clarified in the legend. How is the heatmap sorted?

####The genes displayed here are for potential Gcn4 binding targets that fall within 750 bp around the peaks. The closest available TSS sequences were obtained from the YeasTSS database (PMID:31032841). Read coverage around the TSS of the target genes encoded on the positive strand and the negative strands were calculated separately and coverage matrix was generated for 1kb upstream and downstream of the TSS of the target genes and heatmap was generated without clustering the values of the columns. The ‘computeMatrix’ and ’plotProfile’ functions of the deepTools package (PMID:27079975) were used to generate coverage matrix.

The figure legends were edited as indicated below , for better clarity

A. (Top) Density plots showing that most target genes have Gcn4 binding peaks upstream of the Transcription Start Site (TSS) in the ChIP samples (red), whereas no such enrichment of peaks is observed in mock samples (blue). Here, all the genes that fall in the vicinity of 750bp around the identified Gcn4 binding peaks are considered to be target genes. (Bottom) A heat map showing read coverage for Gcn4 binding, including 1kb upstream and downstream of predicted/known transcription start sites (TSS) of target genes. The heat map on the left shows read coverage in IP samples, and on the right shows coverage in mock-IP (control), both from cells grown in MM+Met. Read coverage around 1kb upstream and downstream of the TSS of the target genes were calculated separately for the genes coded on the positive strand and the negative strands, and merged. The heatmap was generated without any clustering of the columns. The ‘computeMatrix’ and ‘plotProfile’ functions of the deepTools package were used for generating read coverage matrix (PMID: 27079975). Also see Supplementary Figure 7 which shows read coverage for Gcn4, in the context of the translation start site (ATG) of each gene.

- Fig 2E: what is the interpretation of the differences between ORF and Non-coding across the different studies? Also the axes on the figures are very different (0-35 for this study vs 0-500 and 0-300) for previous studies. What is the relevance of this? Overall it is not clear what the message of this figure is. Despite the use of "strikingly" in the associated sentence it is not clear to me what this result means.

####Our interpretation is only that the signal between the ORFs and noncoding genes are not very different under starvation conditions. However, under growth conditions, the Gcn4 occupancy is strongly enriched on the promoter. This distinction can be putatively explained because the amounts of Gcn4 (and other transcriptional regulators) under these conditions are likely to be different from conditions where Gcn4 is induced by pharmacologically inhibiting amino acid biosynthesis. In such conditions, high Gcn4 amounts could result in small amounts of promiscuous binding to ORF regions as well. We do not wish to overspeculate on, or overinterpret this point, and will allow readers to come to their own conclusions. We have edited this section to only make a more succinct statement (lines 277-281).

*“These analyses show that during a growth program, or homeostatic adjustments to altering nutrients during growth, the direct targets of Gcn4 remain specific, and restricted to the promoter regions of genes. This contrasts with conditions where amino acid biosynthesis is severely restricted, and where Gcn4 occupancy may extend to ORF regions as well. “*

We have removed ‘strikingly’ everywhere in the manuscript.

- Fig 3A: how does this look for the starvation program? Are the direct genes the same? This is important for interpreting the context-dependent role of GCN4.

\#### The direct targets of Gcn4 under amino acid starvation conditions are also enriched for amino acid biosynthesis pathways, as is also observed for the growth condition (+met). The indirectly activated genes do differ in their biological processes between starvation and growth. Transcripts indirectly activated by Gcn4 under starvation are enriched for several catabolic processes such as carbohydrate catabolism, amino acid catabolic process, while under growth condition indirect targets activated by Gcn4 are typically involved in anabolic processes. This GO grouping of direct and indirect targets of the starvation programme are now listed in the supplementary worksheet WS3.

In the case of repressed genes, nearly all the transcripts involved in cytoplasmic translation are indirectly repressed by Gcn4 in the methionine induced growth program. In the starvation contexts, a few translation related genes appear to be directly repressed by Gcn4. This is included now in the Supplementary worksheet WS3.

Minor note: The published starvation datasets that are available for Gcn4 (which we have used) do not have time and condition matched transcriptomes and ChIP-seq data. Those studies had combined and pooled different data in their analysis. So we have used these datasets cautiously, and do not want to overstate or over quantify our results. Our own datasets (in this study) are time and condition matched, so we can make strong statements only about our data. We have therefore made cautious conclusions about this in the results (lines 341-345), and can only say that the Gcn4 targets are well conserved irrespective of the growth state of the cell.

- Fig 3E/F, similar comment to above - instead of simple overlaps the authors should show scatterplots of fold-changes to allow readers to interpret the full data.

\#### We now also include scatterplots in Supplementary Figure 11, in addition to the overlaps shown in the main figure. This is also indicated below (with an explanation), for convenience.

Note: The magnitude of binding under starvation conditions is higher than the starvation condition. This can be possibly explained by the varying amounts of Gcn4 protein under these very different modes of induction. Unfortunately, none of the published studies show the amounts of Gcn4 protein present, concurrent with the genomic data provided. So a very direct comparison (and more extensive conclusions) are not possible.

- Fig 4: similar to above - show the starvation response GCN4 targets a similar enrichment? I.e. is this mechanism conserved across different cellular context?

\##### Expectedly, under starvation conditions, the transcripts repressed by Gcn4 are also enriched for translation related processes and they are rich in arginine and lysine composition. Similar to the growth condition, there is a significant enrichment of the Gcn4 targets (repressed by Gcn4) in bin3. This suggests that irrespective of the growth context, genes repressed by Gcn4 are rich in arginine and lysine codons. The above points and the new supplemental Figure 13 now clarify this point, and this is indicated in the revised text as well (lines 387-396).
